# Influence of Zn^2^⁺ Concentration on Ceramic Coatings for Corrosion Protection of Magnesium-Lithium Alloys

**DOI:** 10.3390/ma18092072

**Published:** 2025-04-30

**Authors:** Yifei Wang, Chunming Liu, Hongzhan Li, Zhen Zhang

**Affiliations:** 1 School of Materials Science and Engineering, Northeastern University, Shenyang 110819, China; wyf860524@163.com; 2 Northwest Institute for Non-Ferrous Metal Research, Xi’an 710016, China; zhangzhen_chd@163.com; 3 Rare Mental Materials Surface Engineering Technology Research Center of Shaanxi Province, Xi’an 710016, China

**Keywords:** magnesium-lithium alloy, plasma electrolytic oxidation, ZnF_2_, corrosion resistance

## Abstract

This study investigates the enhancement of corrosion resistance in magnesium-lithium alloys through plasma electrolytic oxidation (PEO) coatings incorporating ZnF_2_ via in situ synthesis. By adjusting Zn^2^⁺ concentrations (4–16 g/L) in a zirconium salt-based electrolyte, ceramic coatings with tailored ZnF_2_ content, thickness, and porosity were fabricated. The optimal Zn^2^⁺ concentration of 12 g/L yielded a ZnF_2_-rich coating with isolated pores and enhanced densification (inner layer resistance R_i_ = 3.01 × 10^4^ Ω⋅cm^2^), achieving a corrosion current density (i_corr_) of 4.42 × 10^−8^ A/cm^2^ and polarization resistance (*Rp*) of 8.5 × 10^5^ Ω⋅cm^2^, representing a 354-fold improvement over untreated LA103Z. Higher Zn^2^⁺ concentrations (16 g/L) induced interconnected pores and ZnO formation, degrading corrosion resistance. Long-term immersion (168 h in 3.5 wt% NaCl) confirmed the durability of Zn_12_ coatings (mass loss: 0.6 mg), while Zn_4_ and Zn_16_ coatings exhibited severe localized corrosion. The study demonstrates that balancing Zn^2^⁺ concentration optimizes ZnF_2_ passivation and pore isolation, offering a scalable strategy for Mg-Li alloy protection in corrosive environments.

## 1. Introduction

The magnesium-lithium (Mg-Li) alloy is presently recognized as the lightest structural metallic material exhibiting a high specific strength [1,2,3]. This alloy demonstrates promising application potential in various domains necessitating weight reduction, including aviation, aerospace, weaponry, the automotive industry, and digital products. Nevertheless, Mg-Li alloys are infamously characterized by their inadequate corrosion resistance, which constitutes a significant bottleneck for their widespread applications. The susceptibility to corrosion is widely acknowledged as a pervasive challenge affecting all magnesium alloys, attributable to their elevated chemical reactivity and less compact oxidation films [1,4,5,6]. Furthermore, the fact that lithium exhibits greater chemical reactivity than magnesium renders Mg-Li alloys even more susceptible to corrosion compared to other magnesium alloys, primarily due to the formation of microscale galvanic cells [7,8,9].

In contemporary times, a variety of surface treatment technologies have been developed, including anodizing, vapor deposition, conversion films, and electroplating, aimed at enhancing the corrosion and wear resistance of Mg-Li alloys [10,11,12]. Plasma electrolytic oxidation (PEO) is an extensively employed surface treatment technique utilized to generate an oxide layer on valve metal surfaces, thereby enhancing their corrosion resistance [13,14]. The most notable advantage of PEO coatings lies in the formation of the oxide film under conditions of elevated temperature and pressure on the substrate surface, which results in superior adhesion of the film compared to other surface treatment technologies, such as vapor deposition and spraying [15]. Nevertheless, although conventional PEO coatings exhibit some degree of corrosion resistance, their protective properties may prove inadequate under extreme environmental conditions, potentially resulting in substrate corrosion. The introduction of reinforcing phases into the coating is essential for enhancing its corrosion resistance [16,17]. Thus, the content of these reinforcing phases is of paramount importance.

Prior research has demonstrated that the modulation of the reinforcing phase content can be effectively achieved by adjusting the concentration of nanoparticles within the electrolyte [18,19]. Nevertheless, the introduction of reinforcing phase particles may lead to their aggregation within the electrolyte, resulting in increasingly inhomogeneous interfaces between the reinforcing phase and the ceramic coating, as well as diminished physical bonding, which can adversely impact both corrosion protection and overall bonding performance [20]. The environmental and health impacts of chemicals used in PEO electrolytes, particularly fluorides such as ZnF_2_ and K_2_ZrF_6_, have raised concerns in recent studies. For instance, fluoride-containing compounds are known to pose ecological risks due to their persistence and potential bio-accumulation [21]. In situ synthesis is accomplished by introducing precursors into the electrolyte during the micro-arc oxidation process through solid-state reactions, facilitating the incorporation of the reinforcing phase and mitigating film failure attributed to particle agglomeration [22]. The corrosion resistance of Mg alloys was enhanced by 45.2% through the in situ synthesis of ZrO_2_ achieved by incorporating K_2_ZrF_6_ into the electrolyte [23]. While strategies like in situ synthesis mitigate particle agglomeration issues, the toxicity of electrolyte additives and their disposal protocols remain critical considerations for industrial scalability [24]. These aspects, though beyond the scope of the current study, highlight the need for future investigations into greener alternatives for PEO processes. The incorporation of fluorine compounds into PEO coatings has the potential to accelerate the coating growth rate by promoting spark discharge [25,26,27], while also facilitating the formation of thicker coatings by enhancing the solution’s conductivity and achieving a lower breakdown voltage [28]. The addition of fluoride has been demonstrated to promote coating growth while significantly reducing energy consumption [29,30]. ZnF_2_ is recognized for its ability to enhance the corrosion resistance of alloys, attributed to its excellent chemical stability [31]. Consequently, the in situ synthesis of ZnF_2_ was selected by incorporating Zn(H_2_PO_4_)_2_ into the electrolyte to regulate the ZnF_2_ content within the coating by modulating the concentration of Zn^2+^ in the electrolyte.

In this study, Magnesium-lithium alloy (LA103Z) was used as the substrate, PEO was employed as the surface modification method, and the corrosion resistance and thermal radiation properties of the PEO coatings prepared by adding variable concentrations of Zn(H_2_PO_4_)_2_ (4, 8, 12, 16 g/L) to the zirconium salt electrolyte were researched. Subsequently, the properties of the obtained coatings were comprehensively analyzed and systematically discussed utilizing a range of advanced characterization techniques, including XRD, SEM, EDS, potentiodynamic polarization, and EIS.

## 2. Materials and Methods

### 2.1. Fabrication of PEO Coatings

**Figure 1:**
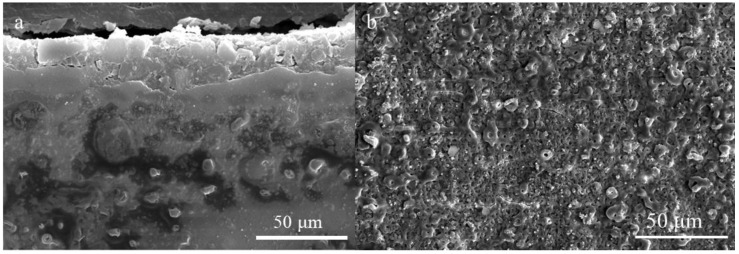
Surface micromorphology and cross-section morphology of Zn0 coatings. (**a**) Surface micromorphology, (**b**) cross-section morphology.

Magnesium-lithium alloys (LA103Z, supplied by Northwest Institute for Nonferrous Metal Research, Xi’an, China) were employed for plasma electrolytic oxidation, with the primary chemical composition detailed in Table 1. Specimens were cut into 20 × 20 × 5 mm blocks using an ATM Saphir 360 precision cutter with flood cooling (cutting speed: 0.1 mm/s, Verder Shanghai Instruments and Equipment Co., Ltd., Shanghai, China). Surface preparation involved sequential grinding with silicon carbide water sandpaper (#200 to #1200, Struers GmbH, Willich, Germany) until achieving Sa ≤ 0.1 μm roughness (measured by Mitutoyo Surftest SJ-410, Mitutoyo Corporation, Tokyo, Japan). Degreasing was performed in analytical-grade acetone (≥99.5%, Sinopharm Chemical, Beijing, China) using an Elmasonic S100H ultrasonic cleaner (40 kHz, 150 W, Elma GmbH & Co. KG, Singen, Germany) for 5 min, followed by triple rinsing in Milli-Q water (18.2 MΩ·cm, Sigma-Aldrich (Wuxi) Life Science & Technology Co., Ltd., Wuxi, China) and drying under compressed nitrogen gas (99.999% purity). The PEO treatment of LA103Z was conducted using a pulsed bipolar power supply. The PEO electrolyte was based on a zirconium salt formulation containing Zn(H_2_PO_4_)_2_, with concentrations of 0 g/L, 4 g/L, 8 g/L, 12 g/L, and 16 g/L. The zirconium salt base electrolyte was composed of F_6_H_8_N_2_Zr6 g/L, H_6_NO_4_P_4_ g/L, and FH_4_N_2_ g/L. For convenience, the prepared coatings are designated as Zn_0_, Zn_4_, Zn_8_, Zn_12_, and Zn_16_. The experimental conditions included a frequency of 1000 Hz, a duty cycle of 15%, and an oxidation time of 15 min. The PEO process was conducted using a combination of constant current and constant potential modes, wherein a constant current density was maintained until the target voltage of 450 V was reached. During the PEO process, the LA103Z specimens acted as anodes, while a stainless-steel plate served as the cathode. The electrolytic cell was kept at a temperature of 25–30 °C using a cryostat to counteract heating of the samples during the process. Finally, the samples underwent ultrasonic cleaning in deionized water, were dried in a vacuum drying oven, and were subsequently stored in a glass desiccator for preservation. Figure 1 presents both the surface micro-morphology and cross-sectional morphology of the Zn₀ coating. It is observed that the coating is nearing delamination, with numerous microcracks on the surface, which suggests poor adhesion between the coating and the substrate.

### 2.2. Microstructural Characterizations

The morphology and microstructure of the fabricated coatings were examined using a scanning electron microscope (SEM, VEGA II XMU, OXFORD, Oxford, UK). The elemental distributions within the PEO coatings were characterized through Energy-Dispersive X-ray Spectroscopy (EDS) microanalysis. The surface porosity percentage was quantified using ImageJ 6.0 software. The crystal structure of the coating was examined by XRD (RIGAKU, Tokyo, Japan) with a step size of 0.02° and a scanning time of 0.15 s per step. XRD diffraction patterns were recorded within a 2θ range of 10–90°. The chemical states within the coating were determined using X-ray photoelectron spectroscopy (XPS, ESCALAB 250Xi, Thermo Fisher Scientific, Waltham, MA, USA) with an Al Kα anode (1486.6 eV). All binding energy values were calibrated according to the adventitious C 1s set at 284.8 eV. The microscopic morphology and three-dimensional structure of the coating surface were observed via a laser confocal microscope (using a Zeiss LSM 800 from Zeiss, Jena, Germany). Additionally, the root-mean-square (RMS) values of the surface roughness for the planar images were quantified utilizing the accompanying software.

### 2.3. Corrosion Test

The corrosion resistance of PEO coatings with varying Zn^2^⁺ concentrations was investigated by performing electrochemical impedance spectroscopy (EIS) and potentiodynamic polarization (PDP) using an electrochemical workstation (VersaSTAT 4, Princeton Applied Research, Ametek, Binghamton, NY, USA). Prior to testing, samples were ultrasonically cleaned in ethanol, dried, and masked with epoxy resin to expose a 1.0 cm^2^ working area. Electrochemical measurements were conducted in a de-aerated 3.5 wt% NaCl solution (300 mL, 25 ± 0.5 °C) using a three-electrode system: a PEO-coated sample (working electrode), platinum foil (20 mm × 20 mm, counter electrode), and saturated calomel electrode (SCE, reference electrode). After 30 min of open-circuit potential (OCP) stabilization (drift < ±2 mV), EIS was performed at frequencies from 10^5^ to 10^−1^ Hz (10 mV AC amplitude, 10 points/decade), followed by PDP scanning from −300 to +300 mV vs. OCP at 0.333 mV/s. EIS data were modeled using ZSimpWin 3.6 with an R equivalent circuit, where Q represents constant phase elements and R corresponds to solution resistance (Rₛ), porous layer resistance (Rₚ), and charge transfer resistance (Rct).

To systematically evaluate the long-term corrosion resistance of the PEO coatings, a 168-h immersion test in 3.5 wt% NaCl solution were conducted. Four parallel samples per group (Zn4, Zn8, Zn12, Zn16) were prepared. Prior to testing, all samples were polished to a surface roughness of Ra ≤ 0.1 μm, ultrasonically cleaned in ethanol, and precisely weighed (±0.01 mg). Immersion tests were performed at 25 ± 2 °C, with the electrolyte refreshed every 48 h to prevent ion accumulation. Post-corrosion, samples were rinsed with deionized water, ultrasonically cleaned (40 kHz, 5 min), vacuum-dried, and reweighed to calculate mass loss. All experiments were conducted using five samples to ensure consistency and reliability of the results.

## 3. Results and Discussion

### 3.1. In Situ Synthesis of ZnF_2_ Nanoparticles

**Figure 2:**
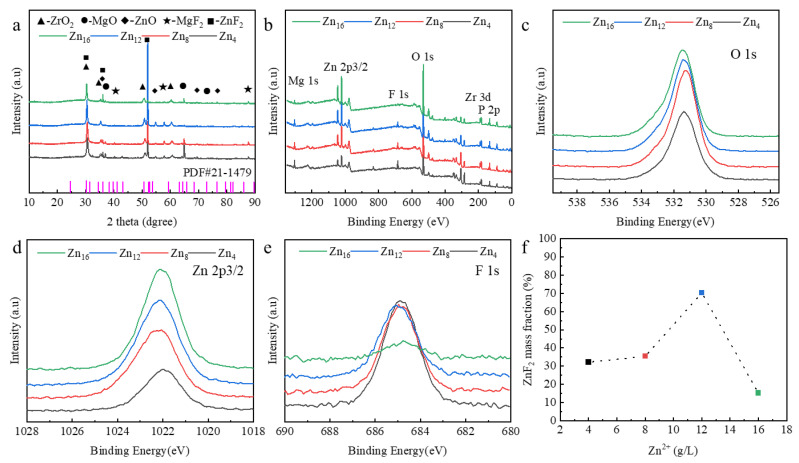
Composition and phase composition of PEO coatings with different Zn^2+^ concentrations: (**a**) XRD, (**b**) XPS survey spectrum, and XPS spectra of PEO coating: (**c**) O 1s, (**d**) Zn 2p3/2, (**e**) F 1s, (**f**) in situ synthesized ZnF_2_ content in PEO coating with different Zn^2+^ concentrations.

Figure 2 presents a comprehensive overview of the characterization results pertaining to the primary components of the nanocomposite coating. The XRD analysis elucidated that the predominant phases within the nanocomposite are MgO, ZrO_2_, and ZnF_2_, as illustrated in Figure 2a. It was observed that an increase in Zn^2^⁺ concentration results in a corresponding increase in the ZnF_2_ content, which subsequently exhibits a decline; conversely, the MgO content displays an inverse trend, initially decreasing before ultimately increasing. The XPS survey spectrum of the PEO coating is depicted in Figure 2b. In addition to the elements Mg, Zn, and O, Zr, F, and P are identified as the principal constituents of the electrolyte. The electron binding energies of Zn, F, and O within the coating were analyzed via XPS examination, thereby confirming the principal components present in the coating, as shown in Figure 2c–e. Furthermore, the peak position of the nuclear energy level corresponding to Zn 2p3/2 aligns with a binding energy of 1022.49 eV, indicating that zinc exists in the 2+ oxidation state [33,34]. Simultaneously, it was observed that the intensity of the characteristic peaks for O and Zn gradually increased with rising concentration, while the behavior of F differed. Notably, the intensity of the characteristic peaks of F 1s reached a minimum at maximum concentration, potentially due to the binding of F^−^ ions to Mg^2^⁺. The ZnF_2_ is synthesized from Zn(H_2_PO_4_)_2_ present in the electrolyte; under the influence of an electric field, zinc precursor (Zn(OH)_2_) particles migrate to the surface of the anode (LA103Z) and adsorb onto the sample’s surface. During the PEO process, the chemical reaction that produces ZnF_2_ is facilitated by the high-temperature environment generated by the intense plasma discharge in the discharge channel. The in situ synthesized content of ZnF_2_ within the coating, as illustrated in Figure 2f, is quantitatively determined using the Rietveld method [35].

At elevated concentrations, the content of ZnF_2_ diminishes, while Zn^2^⁺ predominantly manifests as ZnO, and F^−^ primarily exists as MgF_2_. This phenomenon can be attributed to the fact that during PEO, the presence of oxygen significantly facilitates the formation of ZnO. At elevated Zn^2^⁺ concentrations, the reaction of Zn^2^⁺ with oxygen is prioritized over that with F^−^, resulting in an enhanced production of ZnO [36]. Concurrently, the presence of Mg^2^⁺ in the solution preferentially reacts with F^−^ to form MgF_2_, thereby further diminishing the production of ZnF_2_ [37]. It was also observed that the content of ZrO_2_ remains unchanged with varying Zn^2^⁺ concentrations; thus, the influence of ZrO_2_ can be considered negligible when analyzing the variations in corrosion performance.

**Figure 3:**
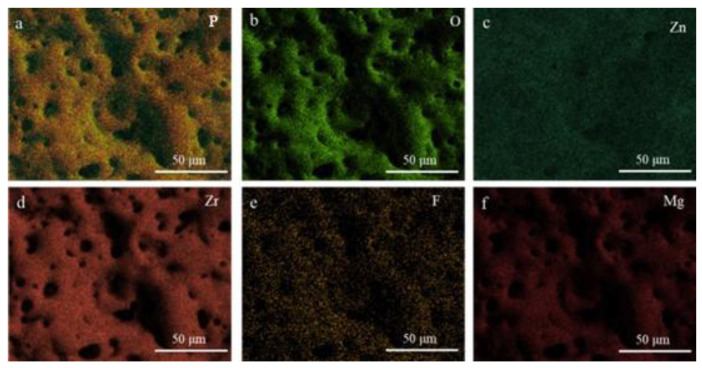
Surface element distribution of Zn_16_ coating: (**a**) P, (**b**) O, (**c**) Zn, (**d**) Zr, (**e**) F, (**f**) Mg.

Figure 3 shows the elemental distribution on the surface of Zn_16_, which shows that a large number of pores are distributed on the surface of the ceramic coating formed by the interconnection of the discharge channels, and the EDS elemental distribution of the coating also shows that the coating is mainly composed of Mg, Zr, Zn, F, and P and that the elements are uniformly distributed in the coating and there is no obvious compositional segregation phenomenon.

### 3.2. Voltage-Time Response During the PEO Process

**Figure 4:**
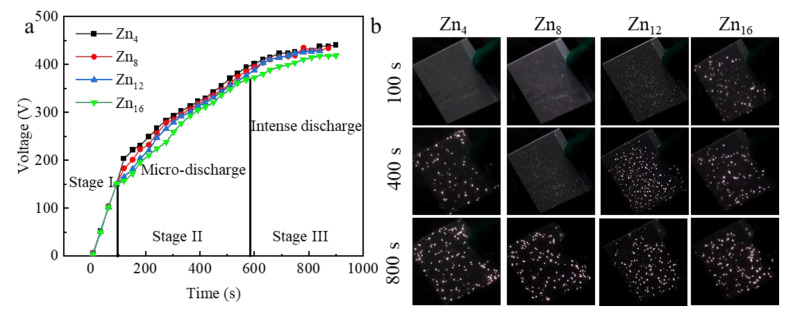
PEO process characteristics: (**a**) voltage-time curves recorded for the PEO process, (**b**) optical photos of spark discharge.

The formation of PEO coatings is a complex process that involves a series of electrochemical, plasma-chemical, and thermochemical reactions [38]. The voltage-time curve consists of both anodizing oxidation and PEO processes (Figure 4a), wherein the PEO process can be divided into three distinct stages. Changes in the arcing voltage were observed with increasing Zn^2+^ concentration, and the lower the arcing voltage, the easier the PEO reaction could proceed, while the arcing time could also reflect the magnitude of the film formation rate at the beginning of the PEO reaction process when the ceramic film was formed. Figure 4b presents plasma discharge images at various stages of the PEO process. Upon reaching the breakdown voltage (Stage I), weak plasma discharges emerge on the specimen’s surface, with the voltage increasing linearly, forming an insulating ceramic passivation film. Stage I is the anodic oxidation stage, where the voltage increases rapidly at a linear rate until dielectric breakdown occurs. At this stage, an initial insulating oxide film is formed on the substrate surface, and a change in the color of the sample surface can be observed due to visible light interference [29]. By Stage II, the appearance of pure white sparks is observed moving across the sample surface. At this point, the surface of the sample is no longer shiny and is dark gray. As the voltage increases, the arc becomes dense, and micro-discharges begin to concentrate at the points of least resistance in the growing oxide layer, such as open porosities, plasma channels, and cracks in the coating, formed in the early stages of the process. Subsequently, a plasma layer forms between the electrolyte and the sample surface [39]. In Stage III, the intensity of the spark discharge increases, leading to the formation of different pore structures within the coating. Intense discharges are observed at 800 s, with significant acoustic emission and gas bursts, indicating an increase in the energy released at the center of the plasma discharge. These aggressive conditions have a deleterious effect on the coating, as the high-energy discharge may lead to pore connections, which increase the pore size, affecting the thickness and roughness of the coating [40].

### 3.3. Effect of Zn^2+^ Concentration on Coating Microscopic Morphology

**Figure 5:**
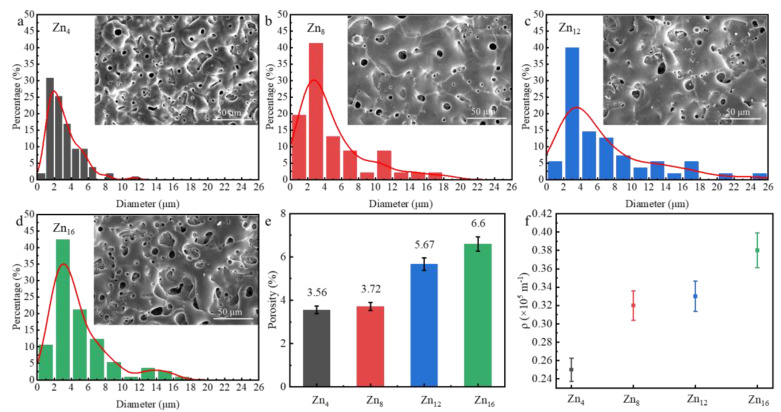
(**a**–**d**) The distribution of pore size for different PEO coatings (the inset shows the surface morphology of different PEO coatings): (**a**) Zn_4_, (**b**) Zn_8_, (**c**) Zn_12_, (**d**) Zn_16_; (**e**) surface porosity of different PEO coatings, (**f**) crack density of different PEO coatings.

The surface morphologies of PEO coatings at varying concentrations of Zn^2^⁺ are illustrated in Figure 5a–d. It is evident that all PEO coatings display porous structures comprising micropores of varying sizes, primarily formed by dielectric breakdown and gas evolution. Dielectric breakdown occurs at coating defects once the applied voltage reaches the critical dielectric breakdown threshold, generating discharge sparks that sharply elevate the instantaneous temperature within the discharge area. Under high temperature and pressure, magnesium and amorphous magnesium oxide from the coating and substrate melt are ejected, forming discharge channels. The intensity of spark discharges is correlated with conductivity, with lower micro-discharge intensities and higher spatial densities observed at various stages of PEO under lower conductivity conditions. As conductivity increases, the power source delivers more energy per cycle, allowing the current to penetrate deeper into the coating, which results in the formation of larger discharge channels and the ejection of greater quantities of molten material. The ejected molten oxide rapidly cool in the electrolyte and subsequently solidify within the discharge channel. The in situ ZnF_2_ within the coating partially deposits in the micropores on the coating surface through adsorption and meshing, while another portion solidifies and swells with reaction products in the molten state of the substrate, forming a micro-convex structure around the discharge channels. This structure is visibly cracked and fragmented around the micropores of the coating.

As the concentrations of Zn^2+^ increase, the pore density decreases, and the pore size increases. Pore size gradually increased from 1 μm to 3 μm. The surface porosity of the PEO coating was increased by 77% when the concentration of Zn^2+^ was increased from 4 g/L to 16 g/L (Figure 5e), which reveals that surface porosity can be altered by regulating the concentration of electrolytes. Quantitative analysis of the crack morphology of the coating using crack density revealed that the crack density on the surface of the coating increased with the increase of Zn^2+^ concentration, and the crack density 0.35 × 10^5^ m^−1^ increased 52% relative to 4 g/L when the Zn^2+^ concentration was 16 g/L (Figure 5f).

**Figure 6:**
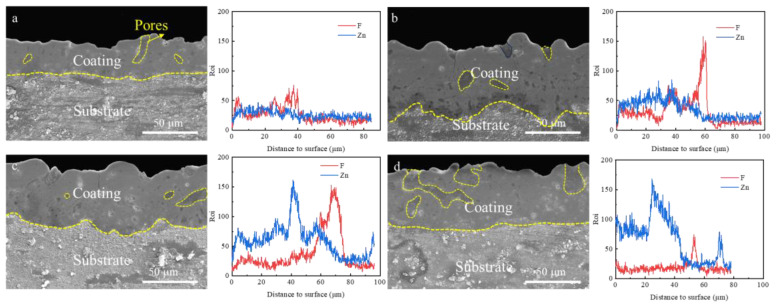
Variation of cross-sectional morphology of different PEO coatings and corresponding Zn and F elements with distance from the surface: (**a**) Zn_4_, (**b**) Zn_8_, (**c**) Zn_12_, (**d**) Zn_16_.

Figure 6 shows the cross-sectional morphology of the PEO coatings at different Zn^2+^ concentrations and the variation curves of Zn and F content with distance from the surface. It was observed that the thickness of the PEO coating tended to increase and then decrease with increasing Zn^2+^ concentration of the coating for the same electrical parameters, and the coating prepared with the addition of 8 g/L Zn^2+^ had the highest thickness. This indicates that increasing the concentration of Zn^2+^ can promote the growth of the coating, but too high a concentration of Zn(H_2_PO_4_)_2_ is not conducive to the formation of the coating. Zn(H_2_PO_4_)_2_, as the main film-forming substance, can directly participate in the growth process of the coating and thus increase the thickness of the coating. At the same time, the increase of Zn^2+^ concentration can reduce the arc starting voltage so that the PEO reaction can be carried out at a lower voltage, which provides a larger energy range for the PEO reaction under the condition of fixed electrical parameters, thus increasing the thickness of the coating. However, a high Zn^2+^ concentration exacerbates the micro-discharge process, and the localized microporous area of the microarc discharge has a high instantaneous temperature, which allows the alkaline electrolyte to dissolve the oxide film more quickly. When the growth rate of the oxide film is lower than the dissolution rate, the thickness of the PEO coating decreases. It was also found that the pore shape and pore volume inside the coating changed significantly with increasing Zn^2+^ concentration. For the Zn_4_ coating, the thickness of the coating was the thinnest, and the connected pores on the coating surface extended from the coating surface to the substrate surface, leading to a decrease in the densification of the coating and weakening of the bond strength between the coating/substrate. With increasing Zn^2+^ concentration, the densification of the Zn_12_ coatings increased, and isolated pores with smaller pore diameters were uniformly distributed inside the coatings with almost no presence of connected pores. However, for the Zn_16_ coating, the further increase in Zn^2+^ concentration resulted in a decrease in the densification of the coating, and connecting pores with large pore sizes could be observed to reappear in the coating. Increase in Zn^2+^ concentration changes the discharge strength increase and leads to changes in coating porosity. The increase in pore size and crack density at higher Zn^2^⁺ concentrations (e.g., 16 g/L) is directly linked to intensified plasma discharge behavior. Elevated Zn^2^⁺ concentrations enhance electrolyte conductivity, leading to higher energy input per discharge cycle. This promotes deeper penetration of discharge channels and violent ejection of molten oxides, resulting in larger interconnected pores (Figure 6d). However, excessive energy input also induces localized overheating (T > 2500 °C), causing thermal stress accumulation and subsequent crack propagation (Figure 6d). The balance between Zn^2^⁺-induced conductivity and thermal stability is thus critical for pore isolation.

**Figure 7:**
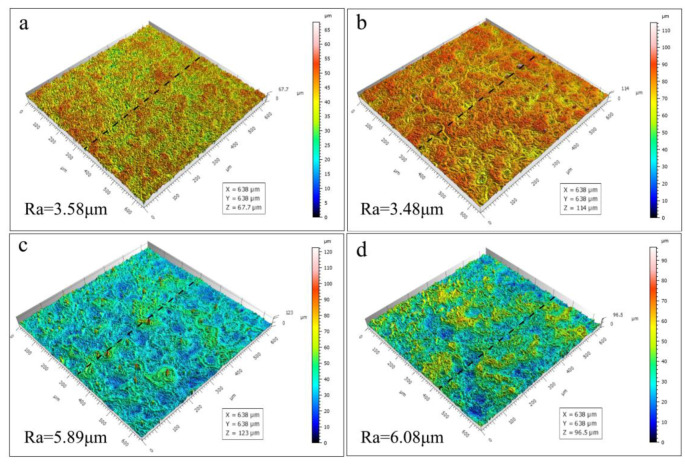
Three-dimensional morphology of different PEO coatings: (**a**) Zn_4_, (**b**) Zn_8_, (**c**) Zn_12_, (**d**) Zn_16_.

The roughness of the PEO coatings was statistically found to exhibit a trend that is not identical to the thickness (Figure 7). With the increase in the concentration of Zn^2+^, the roughness gradually increases; this is because the increase in concentration leads to an increase in the conductivity of the electrolyte, causing an increase in the reaction temperature, which leads to the occurrence of localized ablation in the ceramic coating that has been formed. Despite the reduction in thicknesses of the Zn_12_ and Zn_16_ coatings, the roughness exhibited increases of 64.5% and 69.8%, respectively, in comparison to the Zn_4_ coating. This phenomenon is attributed to localized ablation effects, resulting in the formation of substantial “island-like” projections on the surface of the coatings, which consequently contributes to the observed increase in roughness.

### 3.4. Corrosion Resistance

**Figure 8:**
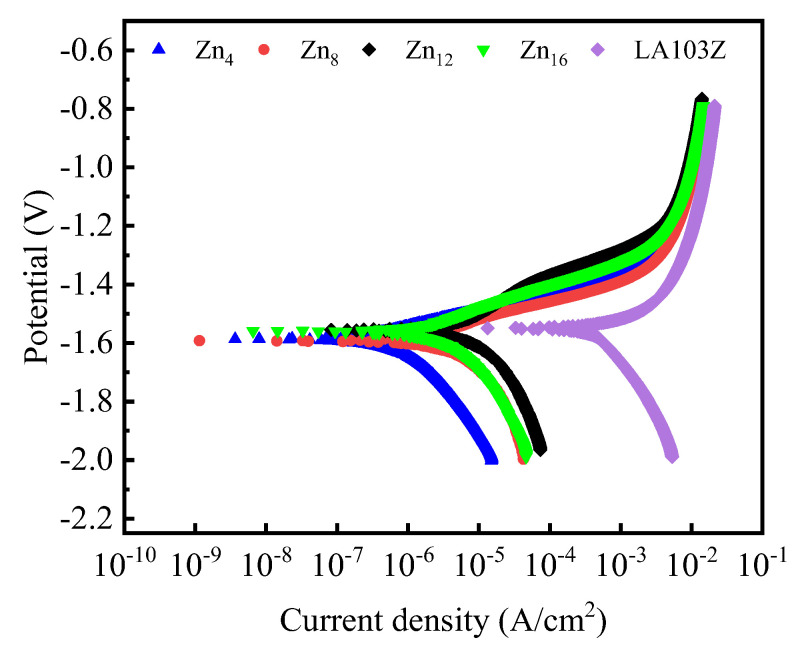
PDP curves of PEO coatings with different Zn^2+^ concentrations.

The potential polarization curves for the PEO coatings are presented in Figure 8. The Tafel extrapolation method was utilized to determine the corrosion potential (*E_corr_*), corrosion current density (*i_corr_*), and the anodic/cathodic Tafel slopes (*β_a_/β_c_*). The polarization resistance (*R_p_*) was calculated using the Stern–Geary equation (Equation (1)), and the results are displayed in Table 2 [41]. The corrosion inhibition efficiency of the various coatings was additionally calculated using Equation (2) [42].

(1) $$R_{p}=\frac{\beta_{a}\times \beta_{c}}{2.3i_{corr}(\beta_{a}+\beta_{c})}$$

(2) $$\eta =\frac{R_{p}^{Zn}-R_{p}^{LA103Z}}{R_{p}^{LA103Z}}$$

It is evident that the corrosion resistance of the ceramic coatings initially increases and then decreases as the concentration of Zn^2^⁺ increases, as reflected by the shift of the PDP curves to the left (lower *i_corr_*) and upward (higher *E_corr_*). Specifically, the Zn_12_ coating exhibited the lowest *i_corr_* (4.42 × 10^−8^ A/cm^2^), the higher *E_corr_* (−1.5499 V), the highest *R_p_* (8.52 × 10^5^ Ω·cm^2^), and the highest η, at 354, which is attributed to the higher ZnF_2_ content in Zn_12_, which effectively fills the micropores and defects, enhances the coating’s densification, and reduces the penetration probability of corrosive media. Additionally, this process reduces the surface reactivity of the substrate and decreases its reaction rate with corrosive media, thereby mitigating the occurrence of corrosion. Moreover, the corrosion inhibition efficiency of all the coatings exceeded 100, which substantially enhanced the corrosion resistance of LA103Z, thereby demonstrating the effectiveness and viability of the coating.

**Figure 9:**
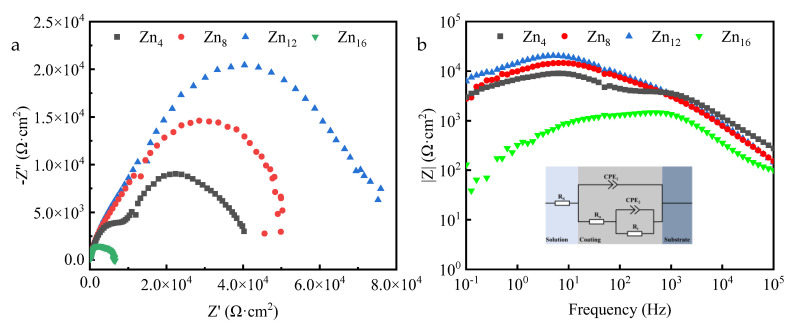
Electrochemical characterization of coatings: (**a**) Nyquist plots, (**b**) Bode plots and equivalent circuit used for EIS spectra fitting (Rs refers to the resistance of the solution from the working and reference electrodes. Ro and CPE_1_ respond to the outside layer’s resistance and capacitance, respectively. Ri and CPE_2_ correspond to the inner layer’s resistance and capacitance.

The EIS spectra (Figure 9a,b) reveal that the impedance semicircle diameter of the ceramic coatings exhibits an increasing and subsequently decreasing trend with the rising concentration of Zn^2^⁺, indicative of its correlation with corrosion resistance. The Bode modulus curves demonstrate that the impedance modulus of the ceramic coatings follows the same trend as that of the impedance semicircle diameter (Figure 9b). The EIS spectra were modeled using an equivalent circuit, and the results are presented in Table 3. Chi-squared values (χ^2^) ranging from 10^−3^ to 10^−4^ were calculated to evaluate the fitting quality. R_s_ represents the resistance of the solution between the working and reference electrodes. R_o_ and CPE_1_ correspond to the resistance and capacitance of the outer layer, respectively. R_i_ and CPE_2_ pertain to the resistance and capacitance of the inner layer. The CPE is characterized as per ref. [43]:

(3) $$Z_{CPE}=\frac{1}{{Y(j\omega )}^{n}}$$

where $Z_{CPE}$ refers to the CPE impedance value, j refers to the imaginary number, ω refers to the angular frequency, while n and Y are CPE parameters. The n value indicates the homogeneity of the coating.

The R(RQ) equivalent circuit (Figure 9b) was selected based on the dual-layer structure observed in Bode phase plots. CPE₁ (outer layer) and CPE_2_ (inner layer) model non-ideal capacitive behavior, where nn-values (0 ≤ n_i_ ≤ 1) reflect coating homogeneity. Higher nn-values (e.g., Zn12: n_2_ = 0.91) indicate near-ideal capacitance, correlating with reduced ionic permeability.

The values of R_o_ and R_i_ for the PEO coatings gradually increase as the concentration of Zn^2^⁺ rises from 4 g/L to 12 g/L. Notably, the Zn_12_ coating displays the highest values of R_o_ (6515 Ω·cm^2^) and R_i_ (30,130 Ω·cm^2^). As the concentration of Zn^2^⁺ increased from 4 g/L to 12 g/L, the values of n_1_ and n_2_ also rose, indicating enhanced homogeneity and capacitance of the coatings, thereby demonstrating that the interface between the substrate and the coating became smoother and flatter. Consequently, the increase in Zn^2^⁺ concentration enhances the corrosion resistance of the PEO coating by reducing porosity and increasing the compactness of the coating, thereby effectively blocking the ingress of corrosive media. The corrosion resistance of PEO coatings is evidenced by the prevention of Cl^−^ penetration from NaCl solutions into the substrate. As the concentration of Zn^2^⁺ increases from 4 g/L to 12 g/L, both the thickness and densification of the coating are enhanced; although surface porosity increases, these are fine, isolated pores that enhance the protective effect on the substrate. Additionally, the Zn_12_ coating contains the highest ZnF_2_ content, which forms a passivation film within the layer to effectively block the infiltration of corrosive media and reduce the reactivity of the metal substrate.

**Figure 10:**
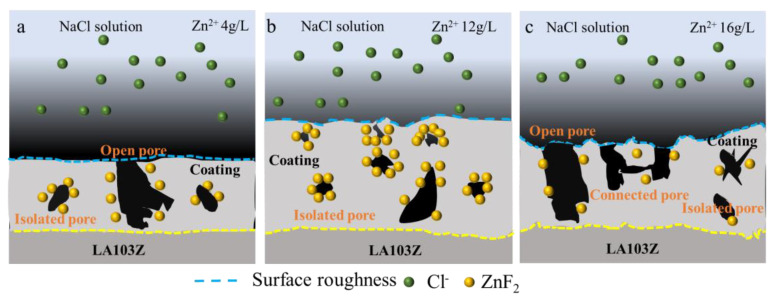
Schematic diagram of corrosion mechanism of PEO coatings. (**a**) Zn_4_, (**b**) Zn_12_, (**c**) Zn_16_.

Figure 10 presents a schematic representation of the corrosion resistance mechanism associated with the PEO coating. Variations in the concentration of Zn^2^⁺ significantly alter the pore morphology and ZnF_2_ content of the coating, thereby impacting its corrosion performance. At a Zn^2^⁺ concentration of 4 g/L, the coating exhibits a reduced thickness, with pores that are directly connected to the substrate. Consequently, the corrosion resistance of the coating is inadequate. When the concentration of Zn^2^⁺ reaches 12 g/L, the coating thickness increases, leading to the predominance of isolated pores. Additionally, this concentration yields the highest ZnF_2_ content, enabling the formation of a complete passivation film on the surface of the coating. At a Zn^2^⁺ concentration of 16 g/L, the heightened conductivity exacerbates electrical discharges, resulting in a decrease in the surface roughness of the coating and the formation of a greater number of open and connected pores. Furthermore, an increase in Zn^2^⁺ concentration facilitates its reaction with O^2−^ to produce ZnO, which in turn results in a reduction in ZnF_2_ content. Consequently, the Zn_12_ sample exhibits superior corrosion performance.

**Figure 11:**
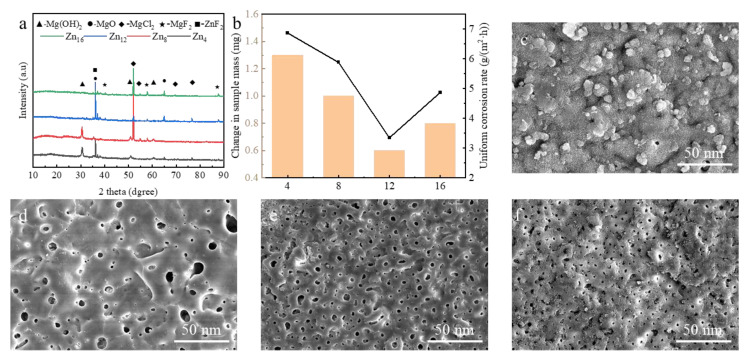
The characterization of PEO coatings after 168 h of corrosion: (**a**) XRD, (**b**) mass loss and uniform corrosion rate, (**c**–**f**) the surface morphology of PEO coatings: (**c**) Zn_4_, (**d**) Zn_8_, (**e**) Zn_12_, (**f**) Zn_16_.

We further evaluated the long-term corrosion performance of the coating through long-term immersion experiments. After 168 h of immersion, Zn4 (Figure 11c) suffered severe localized corrosion, characterized by enlarged cracks and corrosion pits. This aligns with its pre-corrosion interconnected porosity, which facilitated rapid Cl^−^ penetration and substrate attack. The XRD spectrum (Figure 11a) confirmed Mg(OH)_2_ and MgCl_2_ formation, indicating substrate degradation. Zn8 showed partial retention of its ZnF_2_-rich layer but exhibited microcrack propagation due to residual stress (Figure 11d). Notably, Zn12 maintained structural integrity (Figure 11e), with minimal surface etching and no detectable Mg-based corrosion products in XRD, underscoring the effectiveness of its dense ZnF_2_ passivation layer. This correlates with its superior electrochemical metrics, including having the lowest corrosion current density (i_corr_ = 4.42 × 10^−8^A/cm^2^), highest polarization resistance (R_p_ = 8.5 × 10^5^ Ω·cm^2^), and lowest mass loss (Figure 11b). Conversely, Zn16 (Figure 11f) exhibited extensive cracking and pore interconnectivity, reflecting its reduced ZnF_2_ content and ablation-induced defects.

The superior corrosion resistance of Zn12 coatings arises from two synergistic mechanisms: (1) ZnF_2_ passivation: The chemically inert ZnF_2_ forms a dense barrier layer, effectively blocking Cl^−^ penetration. (2) Pore isolation: Isolated pores in Zn12 coatings (Figure 6c) extend the diffusion path of corrosive media, as described by the percolation theory. In contrast, interconnected pores in Zn16 coatings (Figure 6d) create direct pathways for Cl^−^ attack, accelerating Mg(OH)_2_/MgCl_2_ formation (Figure 11a).

## 4. Conclusions

In the present work, PEO coatings with different ZnF_2_ content were prepared by adding Zn(H_2_PO_4_)_2_ combined with PEO in the electrolyte.

1. Zn^2^⁺ concentration critically governs ZnF_2_ content via in situ synthesis. At 12 g/L Zn^2^⁺, ZnF_2_ peaks, preferentially filling pores/cracks to form localized passivation layers. Excessive Zn^2^⁺ (>12 g/L) promotes ZnO formation, reducing ZnF_2_ content and weakening passivation.
2. Despite increasing surface porosity and crack density (0.35 × 10^5^ m^−1^) with Zn^2^⁺ concentration, Zn_12_ exhibited optimal corrosion resistance. This arises from its isolated pore structure, which elongates Cl^−^ diffusion paths, and enhanced inner-layer densification (Rᵢ = 3.01 × 10^4^ Ω·cm^2^), offsetting surface defects.
3. Zn_12_ maintained structural integrity after 168 h immersion, with minimal mass loss (0.6 mg), while Zn_4_ and Zn_16_ suffered severe localized corrosion due to interconnected pores. This work proposes an in situ ZnF_2_ synthesis strategy for Mg-Li alloy PEO coatings, resolving the paradox between porosity and corrosion resistance by emphasizing pore isolation and ZnF_2_ passivation. It provides a novel framework for optimizing high-reactivity Mg alloys in corrosive environments.
4. We propose replacement of toxic Zn(H_2_PO_4_)_2_ with organic zinc salts in future experiments to reduce the ecological risks associated with fluorine. This includes combining nanoparticle doping (e.g., ZrO_2_) or post-sealing treatments to further reduce the porosity and improve the homogeneity of ZnF_2_ and degradation of the coatings under cyclic stress, high temperatures, or acidic conditions for industrial validation.

## Figures and Tables

**Table 1:** Chemical composition of LA103Z magnesium-lithium alloy (wt%) [32].

Element	Li	Al	Zn	Si	Fe	Mg
Content/%	10.0	3.2	2.8	0.05	0.05	Bal

**Table 2:** Corrosion data from potentiodynamic polarization curves of PEO coatings with different Zn^2+^ concentrations.

Sample	*i_corr_* (A/cm^2^)	*E_corr_* (V)	$-\beta_{c}$ (V/dec)	$\beta_{a}$ (V/dec)	$R_{p}$ (Ω·cm^2^)	η
Zn_4_	1.8 × 10^−7^	−1.59	0.14	0.06	2.8 × 10^5^	116.5
Zn_8_	1.1 × 10^−7^	−1.58	0.13	0.38	3.7 × 10^5^	155.3
Zn_12_	4.4 × 10^−8^	−1.54	0.13	0.27	8.5 × 10^5^	354
Zn_16_	1.2 × 10^−7^	−1.55	0.13	0.05	4.1 × 10^5^	169.8
LA103Z	3.9 × 10^−5^	−1.55	0.62	0.33	2.4 × 10^3^	

**Table 3:** Fitting results of EIS plots of PEO coatings with different Zn^2+^ concentrations.

Sample	Rs (Ω·cm^2^)	C_1_ (Ω^−1^ Sn·cm^2^)	n_1_	R0 (Ω·cm^2^)	C_2_ (Ω^−1^ Sn·cm^2^)	n_2_	Ri (Ω·cm^2^)	$\chi^{2}$
Zn4	36.2	1.3 × 10^−5^	0.79	478	4.9 × 10^−5^	0.84	7036	1.78 × 10^−3^
Zn8	40.1	3.8 × 10^−5^	0.86	1932	2.7 × 10^−5^	0.92	20,640	9.68 × 10^−3^
Zn12	42.7	2.3 × 10^−5^	0.85	6515	2.4 × 10^−6^	0.91	30,130	2.71 × 10^−2^
Zn16	36.5	7.0 × 10^−6^	0.82	356	9.3 × 10^−5^	0.84	6157	7.24 × 10^−3^

## Data Availability

The original contributions presented in the study are included in the article; further inquiries can be directed to the corresponding author.
